# Application of Principal Component Analysis Approach to Predict Shear Strength of Reinforced Concrete Beams with Stirrups

**DOI:** 10.3390/ma14133471

**Published:** 2021-06-22

**Authors:** Seungbum Koo, Dongik Shin, Changhyuk Kim

**Affiliations:** 1 The MathWorks Inc., Natick, MA 01760, USA; findseungbum@gmail.com; 2 School of Civil, Architectural Engineering and Landscape Architecture, Sungkyunkwan University, Seoul 03063, Korea; s91120@naver.com; 3 Department of Architectural Engineering, Inha University, Incheon 22212, Korea

**Keywords:** shear strength, reinforced concrete beam, artificial neural network, principal component analysis

## Abstract

The reinforced concrete (RC) member’s shear strength estimation has been experimentally studied in most cases due to its nonlinear behavior. Many empirical equations have been derived from the experimental data; however, even those adopted in the construction codes do not thoroughly and accurately describe their shear behavior. Theoretically explained equations, on the other hand, are aligned with the experiment; however, they are complicated to use in practice. As shear behavior research is data-driven, the machine learning technique is applicable. Herein, an artificial neural network (ANN) algorithm is trained with 776 experiment results collected from available publications. The raw data is preprocessed by principal component analysis (PCA) before the application of the ANN technique. The predictions of the trained algorithm using ANN with PCA are compared to those of formulae adopted in a few existing building codes. Finally, a parametric study is conducted, and the significance of each variable to the strength of RC members is analyzed.

## 1. Introduction

Unlike the bending and compressive behaviors of reinforced concrete (RC), their shear behavior has yet to be theoretically well described. Typical equations that explain an RC member’s bending and compression behaviors begin from decent assumptions and arrive at their final expressions through mathematical derivation. Meanwhile, the equations that describe the shear behavior still predominantly depend on statistical analysis and regression based on laboratory experiments.

For such empirical equations to better predict the behavior of the RC elements, collecting more data and considering a more comprehensive range of input parameters is preferable and can provide more generality. Such prerequisites make research on the shear behaviors of RC members an excellent subject in which to apply machine learning (ML) techniques, as a more extensive data set is typically more beneficial for training a learning algorithm.

The empirical approach has a potential drawback. In contrast to the analytically derived equations, where the dependency on specific variables is apparent, researchers do not know whether a particular variable is a control parameter or not. For this reason, it is considered safer to keep more variables as potential control parameters when experimenting; however, such practice inevitably increases the size of the dataset. The bigger size of the dataset, as a consequence, slows the training speed of a learning algorithm and sometimes even harms the prediction accuracy. This compensation is known as the curse of dimensionality. To overcome such a curse, raw data is preprocessed before being fed into the learning algorithm. Among many candidates of preprocessing techniques, this article specifically focuses on the method called principal component analysis (PCA). It seeks the applicability of PCA to the raw dataset as a means of preprocessing.

PCA can reduce the size of the dataset. PCA can reduce the number of input variables (or, in ML terminology, the features) through statistical analysis of the relationships between the features in the dataset. Although there are many available features in the dataset, not all of them equally affect the behavior of the RC member (or, in ML terminology, the label). When PCA preprocesses a dataset, it returns a set of mutually independent linear combinations of the existing features in the dataset. These processed features are called the principal components.

There can be a couple of strategies once we obtain the principal components. One can select a few components that describe the correlation in the dataset better than other components. On the other hand, one can choose all principal components without benefiting from dimension reduction but only taking advantage of orthogonalization. If a few principal components are selected, the training speed is expected to be faster since the dimension is reduced. Suppose all of the principal components are selected. Although we may not notice any speedup in training, the quality of the result is expected to be better due to the orthogonalization of the input dataset.

Since the early 2000s, researchers in the structural engineering field have begun to adopt ML techniques into their research, particularly to predict the shear behaviors of RC members better. The main learning algorithm considered has been a feed-forward backpropagation artificial neural network (FFBP ANN), where the input variables are selected according to the characteristics of the experiments. Sanad and Saka [1] trained a conventional multilayer FFBP ANN to predict the ultimate shear strength of reinforced concrete deep beams using 111 points of experimental data with ten variables. Their findings showed that ANN returned a reliable prediction even when no single equation existed that accurately describes the ultimate shear strength of RC deep beam. Adhikary and Mutsuyoshi [2] conducted a study to predict the shear strength of a concrete beam that was reinforced by externally bonded steel plates using traditional ANN. In their research, 469 and 85 finite element analysis results were used in total for the training set and test set, respectively. Sucharda [3] conducted a detailed analysis of a reinforced-concrete beam without shear reinforcement, which was based on a complex set of laboratory tests and non-linear analyses with a sensitivity study. Cladera and Marí [4,5] published two separate studies on the shear design procedure of an RC beam: RC beams reinforced with and without the vertical reinforcements, as well as with the traditional FFBP ANN. In total, 123 experimental data points were used in their research on the RC beam with stirrup, with the data set divided into 104 training data and 19 validation data. Mansour et al. [6] suggested an algorithm which predicts the shear strengths of RC beams with 176 experimental data where there are nine input variables. They verified that the prediction by ANN resulted in less error than the empirical equations suggested in the contemporary design codes. Oreta [7] examined the size effect of RC beams without stirrups to their shear strength using a multilayer FFBP ANN. In total, 118 experimental data with five input variables were used, and only five nodes were presented in each of the hidden layers. The trained model could successfully simulate the effects of input variables on the shear capacity of RC beams without stirrups. Lehmann [8] and Wang [9] conducted studies on the shear capacity of reinforced concrete members using fibers. Adhikary and Mutsuyoshi [10], again, trained two ANN models to predict the shear strength of steel fiber RC beams. The numbers of experimental specimens used for the training set and validation set were 70 and 15, respectively. There were four to five nodes in each of the hidden layers, and the depths of the layers were three in both cases. Abdalla, Elsanosi, and Abdelwahab [11] also used the multilayer FFBP ANN technique to predict the shear strength of RC beams. The data set comprised 164 experimental observations, where each observation consisted of six input variables. Yang, Ashour, and Song [12] used 631 experimental data to train a multilayer FFBP ANN with the early stopping technique to predict the shear capacity of deep beams, and 549 observations to do the same for slender beams. The prediction by the trained model was compared to the ACI 318-05 and EC2 specifications, and a case study was also conducted regarding the input variables. Perera et al. [13] used 98 experimental data to train an ANN model for the prediction of the FRP-strengthened RC beams’ ultimate shear strength. 11 input variables were considered, including the variables that represent the effect of FRP, and the ANN model consisted of a single hidden layer with 11 nodes. They also verified the input variable with the greatest effect on the shear strength of FRP-reinforced RC beams. A study by Tanarslan, Secer, and Kumanlioglu [14] used the experimental data of 84 FRP-strengthened RC beams to estimate their shear strength. Nine input variables were chosen to incorporate the effects of concrete, steel reinforcement, and FRP. There was only one hidden layer consisting of four nodes. The trained model was compared to the current design code.

Although there have been many previous studies, the number of experimental data used for training and validation has rarely exceeded 500, and none of them has preprocessed the raw data using PCA to boost the training efficiency and prediction accuracy. The research introduced in this article uses a data set consisting of 776 shear experimental results and secured data from the precedent publications [15,16,17,18,19,20,21,22,23,24,25,26,27,28,29,30]. Although it is not determined how many data points should be utilized to develop an ANN model, the regression results showed a significantly low error percentage, which implies that a sufficient number of data points was used in this study. Eight additional validation sets then validated the trained algorithm, and a parametric study was conducted afterward.

The study on the shear behavior of RC members is data-dependent, and the effectiveness and correlations between the control parameters of the experiment are not foreseeable. Based on such character, this article proposes an ANN approach with 776 PCA preprocessed experiment data [15,16,17,18,19,20,21,22,23,24,25,26,27,28,29,30] to predict the shear behavior of the RC beams with transverse reinforcements. The trained algorithm was then validated using eight additional points of experiment data. Upon completion of the validation, the prediction given by the trained model was compared to the existing building codes: ACI 318-19 [31] and EC2-04 [32], as well as the model proposed by Lee and Kim [33], to determine whether the suggested approach in this article is worthwhile.

## 2. Shear Strength in Design Codes

### 2.1. ACI 318-19

The design equation for the shear strength of reinforced concrete beams specified in ACI 318-19 [31] is as follows:

(1) $$V_{u}\leq \phi V_{n}$$

In this equation, *V_u_* is the factored shear force at a section, $V_{n}$ is the nominal shear strength, and *ϕ* is the strength reduction factor. $V_{n}$ in Equation (1) can be decomposed into the contributions of concrete and shear reinforcements as follows:

(2) $$V_{n}=V_{c}+V_{s}$$

For non-prestressed members the concrete shear contribution, $V_{c}$, shall be taken either of the following.

If $A_{v}\geq A_{v,min}$

(3) $$V_{c}=\left[0.17\lambda \sqrt{f_{c}^{\prime }}+\frac{N_{u}}{6A_{g}}\right]b_{w}d\;\;\mathrm{or}\;V_{c}=\left[0.66\lambda {\left(\rho_{w}\right)}^{1/3}\sqrt{f_{c}^{\prime }}+\frac{N_{u}}{6A_{g}}\right]b_{w}d$$

otherwise

(4) $$V_{c}=\left[0.66\lambda_{s}\lambda {\left(\rho_{w}\right)}^{1/3}\sqrt{f_{c}^{\prime }}+\frac{N_{u}}{6A_{g}}\right]b_{w}d$$

where $b_{w}$ is width of the web, d is effective depth of the section, $\rho_{w}$ is ratio of longitudinal tensile reinforcement, $f_{c}^{\prime }$ is compressive strength of concrete, $N_{u}$ is axial load, $A_{g}$ is gross cross-sectional area of the member, λ is modification factor for lightweight concrete, $\lambda_{s}$ is size effect factor.

The size effect factor, $\lambda_{s}$, is introduced by assuming that the shear strength of members with shear reinforcement less than the minimum required by code, does not increase in proportion to the member depth. This factor can be calculated using the following equation,

(5) $$\lambda_{s}=\sqrt{\frac{2}{1+0.004d}}\leq 1$$

The shear reinforcement contribution is based on the 45° truss model, using the following equation,

(6) $$V_{s}=\frac{A_{v}f_{yt}d}{s}\left(\sin\alpha +\cos\alpha \right)$$

where *A_v_* represents the area of shear reinforcement, $f_{yt}$ specifies the yield strength of shear reinforcement, and α is the smaller angle between shear reinforcements and the longitudinal reinforcement.

*V_s_* in Equation (6) is derived from equilibrium in the 45 degrees truss model. The 45 degrees truss model assumes:

(a) A diagonal crack occurs in the concrete principal compressive stress direction.
(b) A diagonal crack occurs at 45 degrees and is uniformly distributed in the web of concrete.
(c) The tensile strength of concrete on the cracked surface can be neglected. The dowel and interlock actions are also not directly considered.
(d) The stress developed in the transverse reinforcement is equal to its yield stress, and the longitudinal reinforcement does not yield and remains in the elastic range.

The 45 degrees truss model provides a simplified model that helps to easily calculate the shear strength of the beam; however, the model tends to underestimate the shear strength since it fixes the crack angle at 45 degrees.

### 2.2. EC2-04

EC2-04 [32] provides a shear strength equation based on the variable angle truss model, and it requires the design shear strength ($V_{d}$) at the ultimate limit state to be greater than the required shear strength ($V_{u}$). Here, $V_{d}$ can be one of $V_{Rd,c}$, $V_{Rd,s}$ and $V_{Rd,max}$, where each are defined as follows.

$V_{Rd,c}$ is the design shear strength of the RC beam without vertical reinforcement, as computed using the following formula:

(7) $$V_{Rd,c}=\left[\frac{0.18}{\gamma_{c}}k{\left(100\rho_{l}f_{c}^{\prime }\right)}^{1/3}+0.15\sigma_{cp}\right]b_{w}d$$

$\gamma_{c}$ in this equation is the strength reduction factor of concrete materials, *k* is the size effect coefficient, $b_{w}$ and d are the width and effective depth of the beam, where the unit is in millimeters. $\rho_{l}$ is the longitudinal reinforcement ratio and $\rho_{cp}$ is the compressive stress of the cross-section.

When $V_{u}$ is greater than $V_{Rd,c}$, a sufficient amount of transverse reinforcement must be installed, and the corresponding shear capacity of the beam is denoted as $V_{Rd,s}$ and should be computed as follows.

(8) $$V_{Rd,s}=\frac{A_{sw}}{s}zf_{ywd}\cot\theta$$

Equation (8) must not exceed $V_{Rd,max}$, which is calculated as

(9) $$V_{Rd,\max}=\frac{a_{cw}b_{w}z\nu f_{cd}}{\cot\theta +\tan\theta }$$

(10) 1≤cotθ≤2.5

(11) $$\nu =0.6\left(1-\frac{f_{c}^{\prime }}{250}\right)$$

ν is the effective coefficient of concrete compressive strength and z is 0.9d. EC2-04 [32] neglects the contribution of concrete to the shear strength when shear reinforcement exists.

### 2.3. Lee and Kim

The classical shear design equations have been improved by compatibility-aided truss models, such as the modified compression field theory (MCFT), rotating angle softened truss model (RA-STM), and fixed angle softened truss model (FA-STM). These sophisticated theories can predict the shear strengths of the beams more accurately; however, the calculations become more complex as they come to involve additional variables and conditional equations. Nevertheless, calculating the yield shear strength, which is slightly smaller than the ultimate shear strength, is relatively simple. Based on this fact, Lee and Kim [33] suggested a simplified but more accurate equation combining the yield shear strength and strain compatibility condition.

The equation considers the ratio of longitudinal reinforcement, shear span, and the moment at the critical section, and is expressed as follows:

(12) $$V_{n}=\sqrt[{3}]{{\left(\rho_{t}f_{yt}+\lambda \lambda_{s}f_{1}\right)}^{2}\frac{700\rho }{k\left(a_{v}/d+1\right)}}b_{w}jd$$

where $\rho_{t}$ is the ratio of transverse reinforcement, $f_{yt}$ is the yield stress of transverse reinforcement, λ is the modification factor reflecting the reduced mechanical properties of lightweight concrete (0.75 for the light weight, 1.0 for the normal weight), $\lambda_{s}$ is the size factor ($\sqrt{200/d^{3/2}}$), *f*_1_ is the principal tensile stress of concrete, ρ is the longitudinal tensile reinforcement ratio, *k* is the modification factor reflecting the effective principal compressive strain of concrete $\left(\beta_{s}\epsilon_{2}{\left(1+\rho_{t}f_{yt}/\beta_{s}f_{c}^{\prime }\right)}^{0.9}\right)$, $a_{v}$ is the shear span, d is the effective depth of the cross-section, $b_{w}$ is the web width, and jd is the flexural lever arm.

## 3. Development of ANN Model

### 3.1. Principal Component Analysis

Instead of directly using the experimental data to train the machine learning algorithm, it is preprocessed beforehand so as to yield faster training and better accuracy. The method used is called principal component analysis (PCA).

Let a set of experimental data $X^{b}$ be a two-dimensional array consisting of m columns and n rows. Typically, not all of the columns are mutually independent, as they are instead correlated to one another to some degree. Such correlation within the data set is known to have a drawback in terms of generalization when it is used to train the machine learning algorithms; in other words, the trained algorithm only predicts well within the data set used for training, and makes poor predictions with new data [34,35]. In addition, highly correlated data lead to the data redundancy problem [12], since they are similar to each other, not all of them are essential, therefore they are not all necessary to be presented to the learning algorithm in the training step. In order to see the dependencies between the columns and ultimately de-correlate the data, PCA can be used.

PCA is an orthogonal projection of m-dimensional observation on to l-dimensional subspace in such a way that the variance of the projection is maximized [36]. In order to achieve the dimension reduction, one should choose l<m; however, if m is already small, one may only seek to de-correlate the features in $X^{b}$ with l=m.

The typical PCA procedure on a data set $X^{b}\in R^{n\times m}$ is as follows. First, each column in $X^{b}$, i.e., $x_{j}^{b}\in R^{n}$, j=1, ⋯, m must be normalized so that the data have a mean of 0 and a standard deviation of 1.

(13) $$x_{j}^{a}=\frac{x_{j}^{b}-\mathrm{mean}\left(x_{j}^{b}\right)1}{\sigma \left(x_{j}^{b}\right)}$$

Here, $x_{j}^{a}\in R^{n}$ is the normalized column vectors; $\mathrm{mean}\left(x_{j}^{b}\right)$ is the mean of $x_{j}^{b}$, which is a scalar; $1\in R^{n}$ is the vector where all entries are 1; and $\sigma \left(x_{j}^{b}\right)$ is the standard deviation of $x_{j}^{b}$, which is also a scalar. The new matrix $X^{a}=\left[\;x_{1}^{a}\;\cdots \;x_{m}^{a}\;\right]$ possesses identical information to $X^{b}$ but it is normalized column-wise.

Next, the correlation $R^{a}$ is obtained by

(14) $$R^{a}=\frac{1}{n-1}{\left(X^{a}\right)}^{T}X^{a}$$

As $R^{a}$ is a square matrix, eigenvalue decomposition is possible, which brings about m eigenvalues and corresponding eigenvectors:

(15) $$R^{a}=V^{a}\Lambda^{a}{\left(V^{a}\right)}^{T}$$

Here, $V^{a}$ is a matrix that has eigenvectors of $R^{a}$ as its column vectors, and $\Lambda^{a}$ is a diagonal matrix having eigenvalues for its diagonal entries. The algebraic and geometric multiplicities are not strictly considered. The eigenvalues are the variance of the corresponding eigenvector, and multiplying $V^{a}$ by $X^{a}$ gives the principal components of $X^{b}$.

(16) $$X=X^{a}V^{a}$$

The size of X and $X^{b}$ are the same; however, all the columns are principal components, not the input variables, and the significance of each principal component is represented by the corresponding eigenvalue.

One may use all the principal components for the training. Such an approach would not reduce the dimension of the data set; however, each column in the preprocessed data set X is now de-correlated so that the learning algorithm can return a better prediction with a shorter training time. On the other hand, one could choose several significant principal components to approximately represent the raw data $X^{b}$ and reduce the dimension of the input data set without losing too much information. Since the data set $X^{b}$ only has eight input variables, all eight principal components were used to train the learning algorithm.

The data set $X^{b}$ used in this article consists of n=776 experiments [15,16,17,18,19,20,21,22,23,24,25,26,27,28,29,30], where each experiment consists of m=8 variables: width of web ($b_{w}$ (mm)), depth of tensile reinforcement (d (mm)), shear span to depth ratio (a/d), concrete 28-day compressive strength ($f_{c}^{\prime }$ (MPa)), yield strength of tensile reinforcement ($f_{y}$ (MPa)), tensile reinforcement ratio ($\rho_{l}$ (%)), yield strength of transverse reinforcement ($f_{yt}$ (MPa)), and transverse reinforcement ratio ($\rho_{t}$ (%)). Refer to the Appendix A for detailed experiment data.

The first three variables, $b_{w}$, d, and a/d, represent the section property of the RC beam, while the concrete material property is represented by the concrete compressive strength ($f_{c}^{\prime }$), and the reinforcement properties are stored in the last four variables, namely $f_{y}$, $\rho_{l}$, $f_{yt}$, and $\rho_{t}$. The spacing and cross-sectional area of reinforcement are implicitly reflected in the two ρ values.

**Figure 1:**
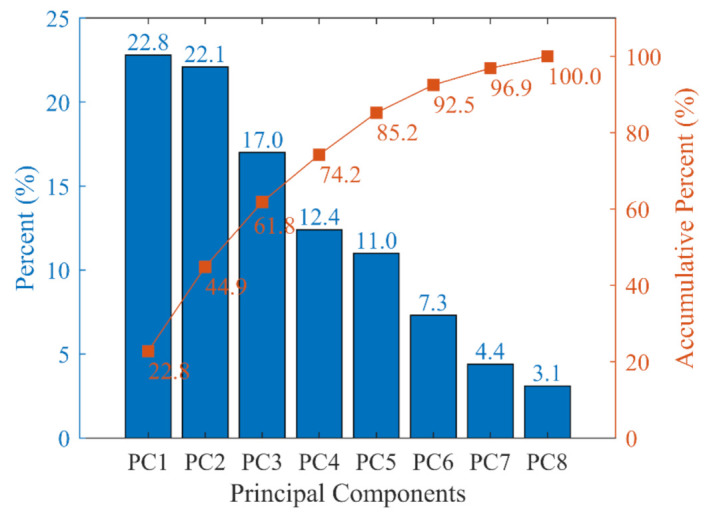
Significance of each principal component (blue bars) and the accumulative importance (red curve).

Performing PCA on this data set yielded the following Scree plot for each principal component, shown in Figure 1. Since there are only eight principal components, all eight are selected in this research to benefit only the de-correlation by PCA and not reduce the dimension. The coefficients of the principal components, i.e., $V^{a}$ in Equation (16), are presented in Table 1.

### 3.2. ANN Model

**Figure 2:**
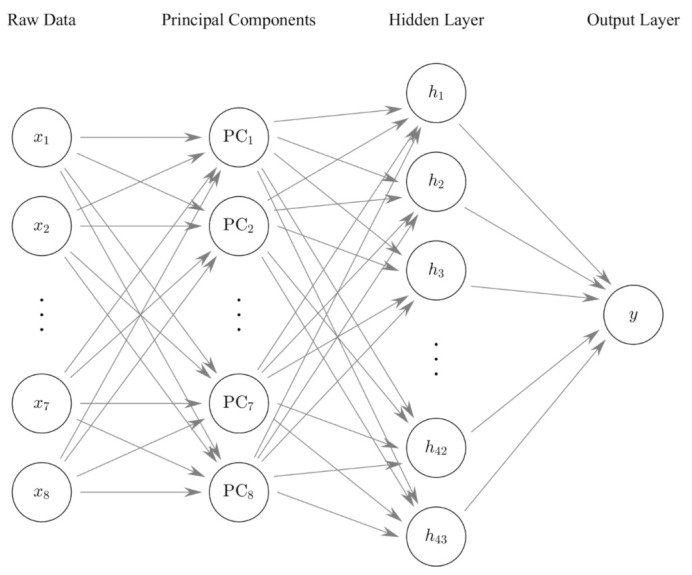
Schematic diagram of FFBP ANN.

The architecture of FFBP ANN used in this research in shown in Figure 2. The preprocessing by PCA is also drawn as a layer of the architecture. The numbers of nodes in the raw data layer and the principal components layer are eight in both cases, since we are using all principal components after preprocessing. There is a single hidden layer, and the number of nodes in the hidden layer is set to 43, which turned out to predict the shear strength with minimal root mean square error (RMSE).

In order to assess the prediction by ANN, three additional measures were calculated: relative absolute error (RAE), root relative square error (RRSE), and the correlation coefficient of the prediction to the experimental measurements.

(17) $$\mathrm{RMSE}=\sqrt{\frac{1}{n}\sum_{i=1}^{n}{\left(\hat{y_{i}}-y_{i}\right)}^{2}}$$

(18) $$\mathrm{RAE}=\frac{\sum_{i=1}^{n}\left|{\hat{y}}_{i}-y_{i}\right|}{\sum_{i=1}^{n}\left|{\bar{y}}_{i}-y_{i}\right|}$$

(19) $$\mathrm{RRSE}=\sqrt{\frac{\sum_{i=1}^{n}{\left({\hat{y}}_{i}-y_{i}\right)}^{2}}{\sum_{i=1}^{n}{\left({\bar{y}}_{i}-y_{i}\right)}^{2}}}$$

where n is the amount of data in the data set, ${\hat{y}}_{i}$ is the prediction, $y_{i}$ is the value in the data set, and $\bar{y_{i}}$ is the mean of ${\hat{y}}_{i}$.

To construct and compare the errors, the model was validated using a 10-fold cross-validation technique. The training data was arbitrarily divided into ten groups (one validation set and nine remaining training sets). The final ANN model utilized all the training data. Eight RC test points were excluded from the training set to spare for performance evaluation of the finalized ANN model.

The accuracy of the predictions of the trained algorithm along with those of the shear strength equations suggested in the three different design codes introduced in Section 2 are measured using the above-mentioned errors; Equations (17)–(19); and the correlation coefficient, and these are summarized in Table 2. As can be seen in Table 2, the RMSE of ANN with PCA is 57.0 percent lower than that of ANN without PCA. Additionally, the average of RAE and RRSE of ANN with and without PCA was 7.1 percent and 16.7 percent, respectively. The prediction by ANN turned out to be the most accurate, followed in order by the results of Lee and Kim [33], then ACI 318-19 [31], and EC2-04 [32] was the least accurate.

**Figure 3:**
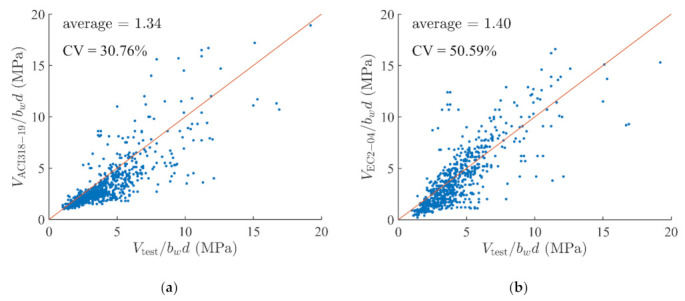

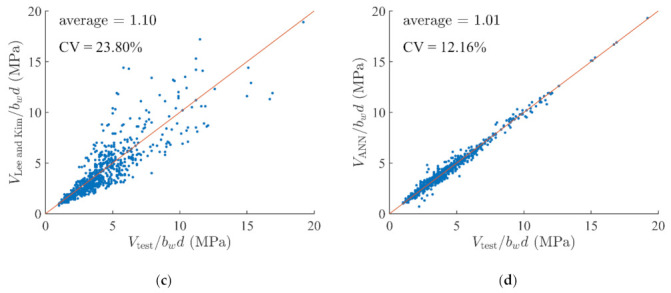
Comparisons between prediction and actual measurements. *x*-axis represents the prediction and *y*-axis presents the measurement. Average is the average of *V*_test_/*V*_code_ whereas CV (coefficient of variance) is calculated as standard deviation of *V*_test_/*V*_code_ divided by the average of *V*_test_/*V*_code_. The subscript, code, for each figure is (**a**) ACI 318-19, (**b**) EC2-04, (**c**) Lee and Kim, and (**d**) ANN with PCA.

Figure 3 shows comparison plots between the actual test results and the predictions by the four different methods mentioned above. The x-axis represents the normalized actual test results while the y-axis represents the normalized prediction by the corresponding method. The red solid line stands for the line y=x. When the prediction is more accurate, data points are gathered nearer the red line. If the dots tend to reside below the red line, the actual shear strength is stronger than the prediction, and we can conclude that the prediction is conservative.

ACI 318-19 [31] (Figure 3a) falls into this case, where the dots are mostly distributed below the red line, therefore ACI 318-19 [31] guides the designers to conservatively design the shear capacity of RC beams. EC2-04 [32] (Figure 3b) also underestimated the shear strength of the given RC beam; however, the trend slightly overshoots when the shear strength exceeds 5 MPa. This is because EC2-04 [32] uses a different formula depending on the shear force that is expected to develop at the critical sections (Equations (7) and (8)). These two design codes are intended to have higher experimental results than the design equations to conservatively design the shear strength and provide simplified equations for the designers. The equation presented by Lee and Kim [33] (Figure 3c) has the dots closer to the red line than the former two. It neither underestimates nor overestimates the shear strength. Finally, the prediction by the ANN model trained with the PCA preprocessed data set has the dots packed very close to the red line, indicating that the prediction is very accurate, as well as the most accurate among the four methods.

It must be noted here that there is a philosophical difference between the equations in the design codes and the equations for the analysis purpose. ACI 318-19 [31] and EC2-04 [32] are design codes, and their purpose is to provide a reliable lower bound that the designers are safe to follow. Since the values are the lower bound, they are born to be conservative and underestimate the real shear capacity of the beams that they are supposed to be assessing. In the meantime, the equations for the analysis are developed to reveal the actual capacity of the beam. Their purpose is to estimate the shear capacity of the member under investigation accurately and as is. The result of the analysis equations is, therefore, not biased by nature. It is evident when we see the experiment data points in Figure 3a,b as the school of blue dots (data points) mostly reside below the solid red line (underestimation of the design code), whereas Figure 3c,d have the blue dots almost equally distributed above and below the solid red line. However, we must also notice that although the analysis equations show no apparent bias in the estimation, the prediction by ANN has less variance than that of another analysis purpose equation by Lee and Kim [33] (Figure 3c). The variance of ANN is also less than two designed codes (Figure 3a,b) from which we can conclude that the prediction by ANN is more accurate than the others regardless of the development purpose.

## 4. Model Validation

### 4.1. Validation Set

The test data of the total eight reinforced concrete beams under monotonic loading was additionally collected aside from the training set to use for the validation set. In Table 3, details of the specimens are shown with the experiment results. This experiment kept the dimensions of the cross-section, concrete strength, yield strength, and the reinforcement ratio of longitudinal reinforcement as constant (control variables) throughout the specimens. The test variables were the yield strength and the transverse reinforcement ratio. The authors must note here that the control and test variables are the purpose of the collected experimental data and have nothing to do with the object or intention of this ANN model’s validation. The training set and this validation set are all amalgamated from the reachable sources by the authors. The current set was selected as a validation set among the data sets where the size of the set is small. In addition, the yield strength and the reinforcement ratio of the transverse bar are known to be the most effective factors that affect the shear capacity of the beam, the current set is more than eligible as a validation set. Moreover, the training set was divided into ten small subsets, and the ANN model was iteratively trained by using nine subsets and validated by one leftover subset to more effectively utilize the training set.

**Figure 4:**
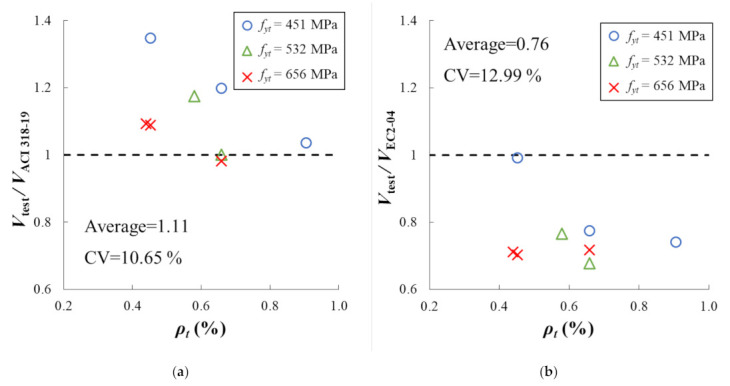

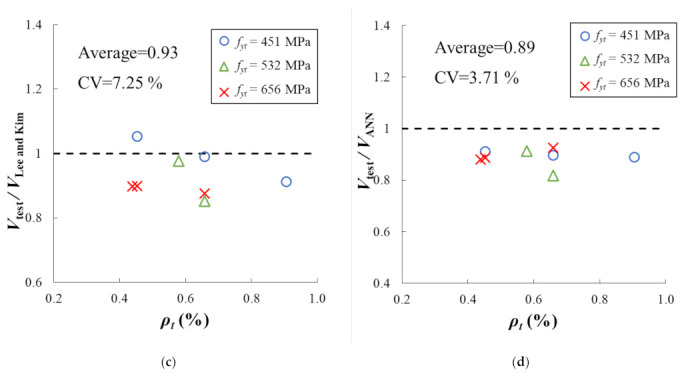
Validation of the four methods. The accuracy is assessed by the distances between the markers and the horizontal dashed line. (**a**) ACI 318-19; (**b**) EC2-04; (**c**) Lee and Kim; (**d**) ANN with PCA.

The ultimate shear strengths of the specimens were calculated using two design codes of ACI 318-19 [31] and EC2-04 [32], the proposed equation by Lee and Kim [33], and the ANN model. Figure 4 shows the validation results of these four methods. The x-axis represents the transverse reinforcement ratio, while the y-axis represents the ratio of measured shear to calculated shear. In the figure, the dashed horizontal line indicates y = 1.0, where the measured value and the predicted value are identical to each other. Different markers are used to differentiate the yield strength of transverse reinforcement in the different test settings. The circular, triangular, and cross markers in the figure indicate the yield strengths of 451, 532, and 656 MPa, respectively. For numerical comparisons, the mean and coefficient of variation are calculated and presented on the corresponding plot.

If the equations or the ANN model predict the shear strength of the RC beam correctly, the markers will be near the dashed line and form a horizontal line. In the cases of ACI 318-19 [31], EC2-04 [32], and Lee and Kim [33], the shear strength ratios tend to decrease as the transverse reinforcement ratio increases, while this trend is not observed for the ANN model. This implies that the ANN considers the effect of the ratio and yield strength of transverse reinforcement, while the other three do not.

ACI318-19 [31] tends to underestimate the shear capacity of the beams compared to those of the other design methods, as mentioned earlier in this section. By contrast, EC2-04 [32] overestimated the shear strength. EC2-04 [32] calculates the crack angle assuming that the concrete and reinforcement reach a plastic state when the reinforced concrete member reaches failure. In the case of plasticity theory, the shear strength can be calculated relatively simply by using only the force equilibrium condition. However, the actual shear strength can be overestimated since it is assumed that the material reaches the plastic state when the member failed. The shear strength was well predicted by Lee and Kim [33] in terms of the mean values. However, the coefficients of variation of ACI 318-19 [31], EC2-04 [32], and Lee and Kim [33] are greater than those of ANN. The coefficient of variation of ANN is calculated to be 3.71%, while they are calculated to be 10.65%, 12.99%, and 7.25% for ACI318-19 [31], EC2-04 [32], and Lee and Kim [33], respectively. According to the results of the validation, we conclude that the ANN predicts the shear strength of the RC beams most accurately.

### 4.2. Parametric Study

**Figure 5:**
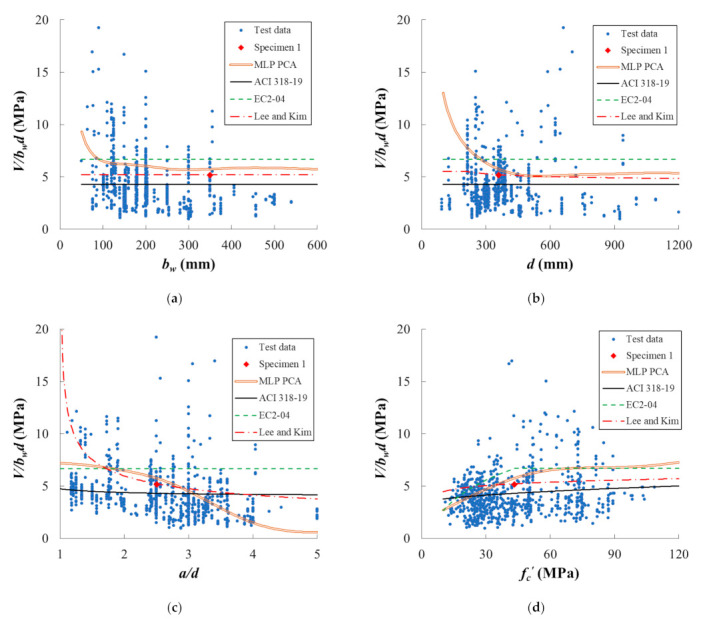

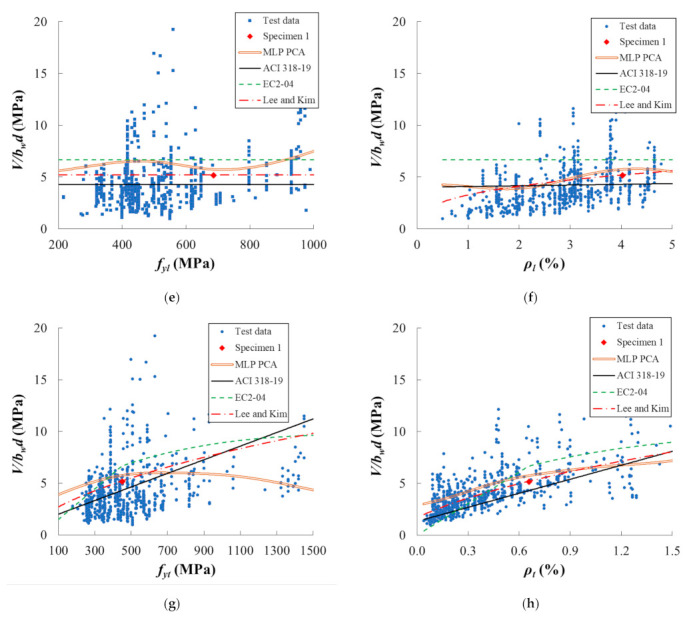
Parametric study. A total of eight variables were varied one at a time while the other variables were fixed. (**a**) Beam width; (**b**) effective depth; (**c**) shear span; (**d**) concrete strength; (**e**) longitudinal reinforcement strength; (**f**) longitudinal reinforcement ratio; (**g**) transverse reinforcement yield strength; (**h**) transverse reinforcement ratio.

Based on the validation data set, a parametric study was conducted on the various input variables that could affect the RC beam’s shear strength. The concrete beams used in the parametric study had the same cross-section and material details as those of specimen 1 listed in Table 3. In order to analyze the effect of individual variables on the shear strength, all of the other variables were fixed while the variable being analyzed was varied along the range of interest; the result is plotted in Figure 5.

The x-axes in Figure 5 are the parameters being analyzed while the y-axis is $V/\left(b_{w}d\right)$, that is, the shear stress developed in the beam. The blue dots represent the data set used to train the ANN model while the pentagram is specimen 1 in the validation set. The yellow dotted curve is the trained ANN model with the preprocessed test data, and the solid purple, dashed green, and dash-dot light blue lines are the three shear strength equations suggested by ACI 318-19 [31], EC2-04 [32], and Lee and Kim [33], respectively.

#### 4.2.1. Beam width and Effective Depth

This subsection discusses Figure 5a,b. The shear behavior of RC beams is affected by the size effect, and the main factors influencing the size effect are the effective depth of cross-section. In the case of ACI 318-19 and EC2-04, the shear stress according to the effective depth was constant because the size effect was not considered. Proposed equation by Lee and Kim reflects the size effect. Therefore, as the effective depth increases, the shear stress tends to decrease gradually. On the other hand, ANN tends to overestimate the shear stress when the effective depth is small. The size effect was not taken into account in the input variables of the machine learning algorithm. This is because the size effect is a dependent variable according to other independent variables, and too many input variables can complicate the algorithm. In order to optimize the algorithm for generalization and simplicity, only essential variables were considered.

#### 4.2.2. Shear Span Ratio

The discrepancy between ANN and the other three equations is apparent in Figure 5c. ACI 318-19 [31] and EC2-04 [32] do not consider the effect of shear span ratio directly, and they appear to be flat lines again, while the experimental data indicates the shear strength is affected by the shear span ratio. The smaller the shear span to depth ratio, the larger the concrete compression zone formed between the support and the loading point, and the greater the shear resistance. On the other hand, when the shear span to depth ratio is greater than 2.5, the shear resistance gradually decreases as the shear span to depth ratio increases. ANN and Lee and Kim [33] predict such shear strength behavior, while the predictions of Lee and Kim [33] rapidly increase when the shear span ratio becomes smaller.

#### 4.2.3. Concrete Compressive Strength

In Figure 5d, all four of the methods predicted that the shear strength would increase as the concrete shear strength increases. Among them, ACI 318-19 [31] and Lee and Kim [33] expected the increment to be linear, while EC2-04 [32] and ANN expected it to be bilinear. For EC2-04 [32], the shear strength was expected to increase until $f_{c}^{\prime }$ reached 40 MPa, while the bilinearity occurred at $f_{c}^{\prime }=60$ MPa for ANN.

#### 4.2.4. Strength of Longitudinal Reinforcement

The strength of longitudinal reinforcement, $f_{y}$ in Figure 5e, turns out to not have a major effect on the shear strength of the RC beam. The experimental data show no significant tendency with respect to $f_{y}$. The models other than ANN do not reveal the effect of $f_{y}$ either and appear to be a flat line, while ANN shows a slight sinusoidal-like curve. The variance of shear strength between $f_{y}$ equals 400 MPa and 600 MPa appears to be large, and this is presumably the effect of other variables.

#### 4.2.5. Longitudinal Reinforcement Ratio

The longitudinal reinforcement (Figure 5f) is closely related to dowel action. Since the longitudinal reinforcement resists shear force acting in the vertical direction, when the amount of the longitudinal reinforcement increases, the shear resistance by dowel action increases. In addition, when the longitudinal reinforcement ratio is large, the width of the crack decreases, which increases the maximum value of the shear component. The experimental data also indicate that the shear strength will increase as the longitudinal reinforcement ratio increases. Both ANN and Lee and Kim [33] capture the effect of longitudinal reinforcement ratio, and the resulting curves behave almost identically. However, ACI 318-19 [31] and EC2-04 [32] did not show any variation per the change in longitudinal reinforcement ratio.

#### 4.2.6. Strength of Transverse Reinforcement

In Figure 5g, the experimental data show higher shear strength for higher strength of transverse reinforcement, $f_{yt}$, but the increment diminishes. ACI 318-19 [31] and Lee and Kim [33] predicted linearly increasing shear strength, while EC2-04 [32] and ANN predicted a diminishing increment. The ANN prediction even expected the shear strength to decrease in the region where $f_{yt}>800$ MPa. This prediction is realistic, since the shear failure is incurred for other reasons, such as concrete crushing, when high strength transverse reinforcement is used.

#### 4.2.7. Transverse Reinforcement Ratio

Figure 5h shows how the transverse reinforcement ratio affects normalized shear strength. Shear strength is not only affected by the yield strength of shear reinforcement, but also by the shear reinforcement ratio. According to the analysis results, all four of the models expect the shear strength to increase as the transverse reinforcement ratio increases. Among the expectations, ACI 318-19 [31] expected the increment to be linear, while the other three expected the increment to be progressively flattened for a ratio greater than 0.6%. When the maximum rebar ratio is exceeded, the prediction result of ANN is close to the actual result because the failure mode may change for reasons such as concrete crushing.

## 5. Discussions and Limitations

As it is commonly applied to experiment-based studies, the prediction by ANN is neither safe nor accurate where the test data exist sparsely. For example, if the effective depth is less than 200mm, both test points and estimation by other equations tell that the shear capacity is less than 7, while the ANN model predicts its exponential growth (Figure 5b). Such a tendency is also observed in the shear span (Figure 5c). When the shear span is greater than 3.5, the ANN model significantly underestimates the shear capacity of the beam as opposed to the other analysis and design equations. Additionally, in Figure 5f, there are no data points to infer how the shear capacity might behave as the longitudinal reinforcement ratio exceeds 6%. Unlike the other three equations that extrapolate the function, the ANN model predicts a mysterious concavity in that region.

The prediction by ANN appears in the middle somewhere between EC2-04 [32] and ACI 318-19 [31] and flexibly leans toward the more accurate side as the training set directs to eventually yields lower error metrics. This characteristic can provide a more efficient and economical design direction. However, it does not mean that the prediction by ANN always supersedes and in preferable to the other equations. Particularly in a region where there is little experimental data, one should question whether the algorithm’s estimation comports with common sense. Such a region typically falls into where the load condition is severe and deformation is extreme.

## 6. Conclusions

This article trained an artificial neural network (ANN) with a dataset consisting of 776 experimental data. The data were preprocessed by principal component analysis (PCA) for variable orthogonalization (de-correlation). The trained ANN predicts the shear strength of reinforced concrete (RC) beams with transverse reinforcement. When preprocessing with PCA, dimension reduction was not used in this research because only eight variables were in the dataset. The preprocessed data was used to train the neural network. The configuration of the neural network was determined through trial and error while numerous combinations of hidden layers and neurons in the layer were sought; the single hidden layer with 43 neurons returned the lowest root mean square error (RMSE).

The preprocessed data was used to train the neural network. The configuration of the neural network was determined through trial and error, while numerous combinations of hidden layers and neurons in the layer were sought; the single hidden layer with 43 neurons returned the lowest root mean square error (RMSE).

Eight additional experiments were conducted in order to obtain the validation set, and the prediction of the trained ANN model was compared to three different shear design equations: ACI 318-19 [31], EC2-04 [32], and the equation suggested by Lee and Kim [33]. The average ratios of experimentally obtained shear strength to shear strength obtained by equations and the ANN model were 0.89, 1.11, 0.76, and 0.93, for ANN, ACI 318-19 [31], EC2-04 [32], and Lee and Kim [33], respectively. The coefficients of variation were also calculated so as to validate the trained model and the shear design equations: 3.71%, 10.65%, 12.99%, and 7.25% corresponding to ANN, ACI 318-19 [31], EC2-04 [32], and Lee and Kim [33], respectively, were reported. For both measurements, ANN was the most accurate. ANN slightly overestimated the shear strength of the RC beam, while the coefficient of variation turned out to be significantly lower than those obtained by the other three methods.

A parametric study was also conducted in order to determine the effects of the input variables considered in this research. In this way, variables that were generally not considered in the shear design equations could be investigated, and their effectiveness to the shear strength could be studied. In addition, ANN reflected the effect of shear span ratio well while the other models did not. The shear strength tended to decrease when the shear span ratio increased from 2 to 3.5. The effect of concrete compressive strength was also observed. ACI 318-19 [31] and the equation by Lee and Kim [33] could not reflect this variable, while EC2-04 [32] and ANN reasonably incorporated it. The effect of longitudinal reinforcement ratio was clearly captured by ANN and Lee and Kim [33]. ANN and Lee and Kim [33] behaved nearly similarly for the varying longitudinal reinforcement ratio. The maximum yield strength of transverse reinforcement that should be used in the shear strength calculation is determined to not exceed 800 MPa. ANN predicted the shear strength to be decreased for $f_{yt}>800$ MPa, meaning that the vertical reinforcement of yield strength greater than 800 MPa negatively affected the RC beam shear strength.

## Figures and Tables

**Table 1:** Principal components (PC). De-correlated data set X is obtained when this table, $V^{a}$, is multiplied by the normalized data set $X^{a}$, i.e., $X=X^{a}V^{a}$.

Features	PC1	PC2	PC3	PC4	PC5	PC6	PC7	PC8
$b_{w}$ (mm)	−0.097	−0.634	0.216	−0.019	0.097	0.264	0.648	−0.207
d (mm)	−0.019	−0.493	0.270	0.485	0.380	−0.156	−0.506	0.152
a/d	0.037	0.031	0.656	−0.591	0.077	−0.141	−0.015	0.438
$f_{c}^{\prime }$ (MPa)	0.272	−0.277	0.184	0.113	−0.855	0.161	−0.204	0.068
$f_{y}$ (MPa)	0.446	−0.248	−0.232	−0.501	0.247	0.371	−0.393	−0.286
$\rho_{l}$ (%)	0.540	0.195	0.376	0.148	0.055	−0.414	0.132	−0.561
$f_{yt}$ (MPa)	0.328	−0.357	−0.470	−0.139	−0.038	−0.607	0.211	0.333
$\rho_{t}$ (%)	0.563	0.223	0.010	0.333	0.211	0.428	0.256	0.479

**Table 2:** Prediction accuracies.

Error Measures	ACI 318-19	EC2-04	Lee and Kim	ANN without PCA	ANN with PCA
Correlation Coefficient	0.9324	0.9039	0.9388	0.9675	0.9977
Root Mean Square Error	120.1279	138.9059	108.9430	76.8855	20.6553
Relative Absolute Error	41.7351	46.5203	34.1850	16.9734	7.4831
Root Relative Square Error	39.6231	45.8169	35.9339	25.3216	6.8130

**Table 3:** Details of the validation set specimens.

**Specimen**	Section			Concrete	Longitudinal Bar		Transverse Bar		Test
	$b_{w}$	d	***a/d***	$f_{c}^{\prime }$	$f_{y}$	$\rho_{l}$	$f_{yt}$	$\rho_{t}$	$V_{\mathrm{test}}$
	**(mm)**	**(mm)**	**-**	**(MPa)**	**(MPa)**	**(%)**	**(MPa)**	**(%)**	**(kN)**
1	350	359	2.5	43	687	4.03	451	0.66	649
2	350	359	2.5	43	687	4.03	656	0.66	698
3	350	359	2.5	43	687	4.03	656	0.44	579
4	350	359	2.5	43	687	4.03	451	0.91	705
5	350	359	2.5	43	687	4.03	451	0.45	572
6	350	359	2.5	43	687	4.03	656	0.45	589
7	350	359	2.5	43	687	4.03	532	0.66	609
8	350	359	2.5	43	687	4.03	532	0.58	652

## Data Availability

All the research data used in this manuscript will be available whenever requested.

## Appendix A

**Table A1:** Details of the training set data.

Section Properties						Concrete		Longitudinal Bars				Transverse Bars			
*b_w_* (mm)		*d* (mm)		*a/d*		*f_c_^’^* (MPa)		*f_yl_* (MPa)		*ρ_l_* (%)		*f_yt_* (MPa)		*ρ_t_* (%)	
Min.	Max.	Min.	Max.	Min.	Max.	Min.	Max.	Min.	Max.	Min.	Max.	Min.	Max.	Min.	Max.
290	290	278	278	2.88	2.88	49	51	536	536	1.950	1.950	460	460	0.110	0.280
127	127	198	203	2.92	3.92	39	103	421	421	3.191	4.531	324	324	0.491	0.785
127	127	198	216	3.00	4.00	45	88	421	421	2.070	4.540	324	324	0.490	0.780
406	406	345	345	2.65	2.65	29	34	434	434	2.310	2.310	544	549	0.390	0.390
300	300	925	925	2.70	2.88	21	80	550	550	0.505	1.010	508	508	0.079	0.079
75	150	704	1369	3.06	3.41	41	42	500	520	9.400	14.470	500	582	1.676	1.774
240	240	300	1200	2.70	2.88	23	25	426	426	1.257	1.257	442	442	0.147	0.147
155	307	464	466	3.76	3.78	22	27	555	555	1.795	1.800	326	326	0.098	0.195
152	305	457	462	3.95	4.01	23	27	400	400	1.670	2.340	340	350	0.100	0.200
139	139	234	234	2.56	2.56	28	28	819	819	1.043	1.043	437	437	0.407	0.407
125	125	215	215	1.40	2.40	52	73	417	417	3.795	3.795	388	388	0.200	1.800
200	200	351	353	2.95	3.08	50	87	500	500	2.278	2.990	530	540	0.109	1.291
152	203	314	389	1.17	2.43	14	48	321	335	1.630	3.420	331	331	0.340	1.220
169	300	459	925	2.50	2.92	47	75	400	550	0.750	1.360	500	508	0.079	0.160
120	120	260	260	2.11	2.88	20	31	396	452	1.920	2.890	314	387	0.200	0.421
152	152	254	272	3.36	7.20	13	37	618	618	1.460	4.160	269	279	0.140	0.830
178	178	267	267	3.62	3.62	20	63	434	434	2.500	3.300	379	379	0.170	0.170
150	150	285	285	1.73	1.73	51	74	953	953	3.060	3.060	297	902	0.498	1.710
200	200	303	303	3.30	3.30	42	42	0	0	2.990	2.990	0	0	0.630	1.150
120	130	500	624	1.25	2.00	24	80	413	440	1.290	2.417	413	590	0.120	1.514
180	180	360	360	1.18	2.35	28	32	343	368	3.210	3.210	250	1392	0.190	1.130
195	201	302	312	3.21	3.31	38	45	621	621	2.860	2.990	571	571	0.110	0.210
80	80	380	380	3.95	3.95	32	32	491	491	3.348	3.348	491	491	0.707	0.707
178	178	299	310	2.95	3.06	14	38	576	602	1.410	4.531	341	529	0.243	1.603
178	178	381	381	2.50	4.25	13	32	515	515	3.810	3.810	343	454	0.187	1.260
120	120	350	350	3.46	3.46	17	29	400	655	2.992	2.992	320	480	0.377	0.670
180	180	450	450	3.33	3.33	68	68	532	532	3.636	3.636	514	514	0.559	0.559
80	80	470	470	3.09	3.19	27	27	500	500	9.525	9.525	550	550	0.707	0.707
152	305	419	419	2.76	2.76	43	43	445	445	1.820	1.820	283	303	0.212	0.341
305	305	539	539	3.10	3.10	36	72	525	525	2.490	2.490	479	479	0.079	0.159
200	200	360	360	2.42	2.42	37	37	216	886	3.150	3.150	931	931	0.410	0.410
155	155	719	719	2.68	2.68	28	29	468	468	2.866	2.866	605	605	0.324	0.432
220	220	244	264	2.00	4.00	42	42	402	436	2.670	3.600	358	358	0.215	0.323
180	180	360	360	1.76	1.76	21	56	798	798	3.160	3.160	1333	1431	0.150	1.000
250	250	198	542	1.75	3.30	64	89	433	452	1.659	4.468	569	632	0.101	0.262
254	254	456	456	3.87	3.87	13	40	271	406	2.228	2.228	237	372	0.109	0.244
360	360	345	345	2.18	2.62	29	78	396	433	2.010	2.010	600	600	0.094	0.189
200	540	252	1383	2.50	4.00	25	85	525	1068	1.440	6.920	334	750	0.150	3.017
50	160	300	375	3.00	3.50	14	24	390	420	2.793	8.378	314	441	0.313	1.251
120	120	940	940	4.04	4.04	23	58	450	500	8.021	12.533	480	480	0.838	1.257
120	120	540	540	2.78	2.78	23	24	616	623	3.879	3.879	647	674	0.524	0.524
86	86	398	398	2.89	2.89	38	38	400	400	1.036	1.036	645	645	0.230	0.230
200	200	336	336	1.79	1.79	23	66	683	1028	2.880	2.880	679	1028	0.190	1.180
150	150	315	315	2.00	3.00	29	34	361	387	2.080	2.610	355	355	0.235	0.471
76	102	216	305	1.18	1.50	16	23	280	431	1.730	1.940	280	437	0.180	2.450
140	203	235	419	2.77	3.60	21	57	400	400	0.680	3.030	243	426	0.040	0.340
150	150	279	279	3.50	3.50	38	38	641	641	1.891	1.891	310	310	0.212	0.212
140	140	464	495	1.64	1.75	23	33	329	329	3.990	3.990	316	378	0.274	0.274
150	150	298	298	3.16	3.16	22	83	448	448	3.360	3.360	266	303	0.124	0.380
85	85	126	130	2.50	3.10	31	50	530	530	2.000	5.720	600	600	0.170	1.188
200	200	360	360	2.42	2.42	20	33	854	854	2.880	2.880	801	870	0.400	0.890
150	150	310	310	3.00	5.00	58	82	425	450	2.590	4.432	255	255	0.139	0.279
110	110	298	298	3.52	3.52	28	31	442	447	3.834	3.834	273	273	0.609	0.609
280	280	273	273	3.11	3.11	29	33	478	478	2.700	2.700	456	587	0.051	0.287
120	175	278	322	3.24	3.57	36	41	977	990	1.070	3.014	323	366	0.215	0.838
152	152	254	272	3.36	5.05	26	34	618	618	1.460	4.160	269	279	0.140	0.830
64	152	254	267	3.33	7.10	11	50	630	630	4.166	9.413	270	704	0.402	2.153
250	450	430	548	3.00	3.00	20	22	469	746	1.361	3.951	557	560	0.336	0.760
77	77	589	590	4.27	4.27	58	58	514	515	13.554	15.613	515	549	1.195	1.678
152	152	305	305	2.46	3.92	19	29	318	414	1.600	2.660	318	354	0.384	1.132
457	457	762	762	2.91	2.91	70	123	431	472	1.877	2.892	445	445	0.158	0.234
356	457	559	762	2.50	3.00	72	125	431	483	1.649	6.971	407	458	0.082	1.771
200	200	260	260	3.37	3.37	46	48	550	550	3.552	3.552	550	680	0.140	0.559
180	180	232	235	2.50	4.00	39	80	495	543	2.229	3.505	820	820	0.093	0.186
125	125	215	215	2.45	2.45	50	71	410	410	3.771	3.771	366	366	0.589	0.589
150	150	260	260	1.73	1.73	56	64	974	974	3.060	3.060	233	929	0.310	0.840
110	110	298	298	3.52	3.52	23	32	420	457	3.834	3.834	349	397	0.305	0.343
90	90	647	662	2.50	2.56	85	87	560	600	6.136	11.686	600	630	0.677	3.867
76	76	95	132	3.00	5.00	26	29	400	400	1.970	3.950	179	283	0.060	0.600
200	200	360	360	2.42	2.42	32	36	931	931	3.090	3.090	285	1235	0.193	1.208
102	200	305	535	1.12	2.38	16	72	431	538	0.884	5.800	353	570	0.150	0.770
110	110	398	463	2.70	3.14	43	74	499	538	1.230	5.800	353	385	0.476	0.476
300	300	400	400	2.50	2.50	18	18	750	750	2.202	2.202	388	388	0.524	0.524
114	114	257	257	3.56	4.44	17	17	441	441	3.367	3.367	290	438	0.326	0.616
229	457	425	851	3.00	3.00	36	43	482	551	0.995	1.048	482	482	0.084	0.334
127	127	198	203	2.74	3.00	41	109	421	421	3.200	4.540	324	324	0.490	0.813
120	120	255	255	1.90	1.90	139	139	960	960	3.900	3.900	1451	1451	0.327	1.230
375	375	655	655	3.23	3.23	34	85	400	400	2.859	5.360	430	430	0.116	0.443
375	375	655	655	3.23	3.23	36	87	400	400	2.800	2.800	430	430	0.081	0.116

Data from Lyngberg (1976), Johnson and Ramirez (1989), Russel (1990), Al-Musawi (1992), Ahamad et al. (1994), Adebar and Collins (1996), Young-Soo Yoon and Denis Mitchell (1996), Kang-Hai Tan, Fung-Kew Kong, and Hai-Yun Lu (1997), Kong, Paul Y L and Rangan and Vijaya (1998), Angelakos (1999), Ozcebe et al. (1999), Collins and Kuchma (1999), Gonzales (2005), Cladera (2002), González-Fonteboa (2002), Yang and Ashour (2008), and Lee (2018).

**Table A2:** Weights of hidden layer of PCA.

*j*	*i*									Output
	1	2	3	4	5	6	7	8	bias	
1	0.883	0.914	0.313	1.607	0.178	0.621	−0.073	0.522	3.997	−1.822
2	0.531	−1.326	0.500	0.394	0.179	0.705	−0.655	−0.470	−0.309	1.415
3	0.014	−0.012	−0.002	−0.008	0.005	−0.002	−0.005	−0.009	0.238	0.000
4	0.251	−0.418	0.445	−0.053	0.173	−0.355	−0.339	−0.763	0.613	0.662
5	1.265	0.510	−0.813	−0.408	−0.618	−0.256	−0.879	−0.123	2.491	1.316
6	0.704	−0.883	−0.408	0.040	0.202	0.255	−0.739	0.534	0.483	1.164
7	−0.461	1.209	−0.478	0.264	−0.343	−0.285	−0.390	−0.002	−0.078	−1.125
8	−0.521	0.286	−0.237	−0.446	−0.107	0.154	1.806	−0.376	−0.861	−1.547
9	0.290	−0.800	0.317	−0.210	−0.593	−0.330	−0.608	1.051	1.486	−1.464
10	0.037	−0.031	0.000	−0.019	0.013	−0.014	−0.020	−0.030	−0.132	−0.045
11	−0.422	−0.388	−0.758	−0.339	0.423	−0.701	0.867	0.019	−1.216	1.199
12	0.619	1.115	0.956	0.684	0.250	−0.512	−0.578	0.929	1.423	−1.528
13	−0.202	1.907	0.154	−0.286	0.580	0.062	−0.141	0.918	2.382	1.526
14	0.547	−0.307	−0.184	−0.928	−0.124	0.953	−0.150	0.715	−0.090	−1.305
15	0.038	−0.032	−0.003	−0.022	0.015	−0.013	−0.020	−0.031	0.019	−0.046
16	−0.758	−0.485	0.404	0.649	0.383	−0.904	0.663	0.137	1.521	−1.076
17	0.975	−0.333	0.976	0.367	−0.296	1.242	−0.266	−1.200	−2.243	−1.790
18	−1.311	−0.589	0.204	0.083	−0.066	1.398	0.034	−1.143	0.949	1.428
19	0.884	1.140	−0.917	0.854	0.394	0.278	−0.255	−0.645	5.764	−1.979
20	−0.752	−0.504	1.024	−0.774	0.146	0.603	0.803	−0.432	0.888	−1.613
21	−0.918	0.293	0.283	−1.272	0.054	−0.223	1.097	−0.855	−0.904	1.456
22	1.136	−1.124	1.079	0.652	0.472	0.173	−0.425	0.846	3.122	1.633
23	0.096	0.363	−0.064	−0.951	0.438	1.017	0.966	0.365	0.032	1.416
24	−0.025	0.029	−0.007	0.022	−0.014	0.010	0.030	0.034	0.019	0.078
25	−0.002	0.003	−0.001	0.001	−0.002	0.004	0.005	0.004	0.132	0.026
26	−0.566	−0.087	−0.370	−0.607	−0.318	−0.934	−0.039	0.801	−0.768	1.212
27	0.781	−0.652	−0.024	−0.626	1.108	−0.166	−0.839	−0.085	−0.078	−1.271
28	0.834	0.768	0.418	−0.501	−0.780	−1.140	−0.006	−0.105	0.299	0.989
29	0.021	−0.018	−0.001	−0.011	0.008	−0.007	−0.011	−0.016	−0.082	−0.018
30	0.198	0.430	−0.105	0.456	0.008	−0.089	−0.335	0.319	1.603	−0.615
31	1.160	1.592	0.005	0.368	−0.360	0.211	−0.107	0.439	1.745	−1.415
32	−0.019	0.012	0.003	0.004	−0.011	0.008	0.008	0.014	−0.876	0.041
33	−0.177	−0.783	−0.363	−0.949	1.007	−0.008	−0.300	−0.531	0.311	1.032
34	0.158	−0.330	−0.183	−0.571	−0.671	−0.172	0.860	−0.750	0.733	0.862
35	0.137	−0.318	−0.680	−0.447	−0.280	0.219	−0.940	1.172	−0.011	−1.274
36	0.030	−0.026	−0.003	−0.017	0.019	−0.004	−0.016	−0.027	0.158	−0.032
37	−1.383	−0.991	0.008	−0.030	0.633	−0.188	0.742	−1.473	−1.733	−1.543
38	−0.295	0.057	0.607	1.244	−0.767	0.176	−0.155	0.481	0.404	1.442
39	−0.889	0.882	0.698	0.329	−0.137	−0.317	−0.028	0.065	2.247	1.291
40	−1.493	−0.451	1.451	0.272	−0.602	0.491	0.682	−0.587	−1.118	1.507
41	−0.002	0.004	−0.002	0.001	−0.002	0.005	0.005	0.005	0.444	0.030
42	0.776	−0.199	−0.120	0.437	−0.147	−0.646	0.331	−1.160	0.096	−1.042
43	1.282	−0.381	0.017	−0.577	0.037	−0.592	−0.578	1.538	−0.151	1.532
